# Collectanea Medica: Consisting of Anecdotes, Facts, Extracts, Illustrations, &c.

**Published:** 1820-03

**Authors:** 


### COLLECTANEA MEDICA:

CONSISTING OF

ANECDOTES, FACTS, EXTRACTS, ILLUSTRATIONS, &c.

*Relating to the History or the Art of Medicine, and the Auxiliary Sciences.*

Floriferis ut apes in saltibus omnia libant,

Omnia nos itidem depascimur aurea dicta.

### *Observations respecting the Functions of the Cellular Tissue, especially in regard to the Phenomena of some Diseases.*

[BORDEU, *Recherches sur le Tissu Muqueux ou l'Organe Cellulaire*, § xcix.]

"*WHEN an angina disappears and falls on the lungs*," says HIPPOCRATES, "*death must be expected on the seventh day; if this be passed over, a suppuration will be formed.*" (Aph. x. § 5.) "*The matter of the angina is carried inwards, when, having disappeared, it oppresses the lungs, and the patient has difficulty of respiration.*" (Coac. cccxlvii.) It is a long time since that those sentences, for the first time, quite astonished me. I visited a physician (Daban de Pau) who was attacked with an affection of the throat, for which he had himself bled twice in the arm, once in the foot, and then he took purgatives. I treated the patient like a very young physician treats another fifty years old. The throat after this appeared to become well, when the patient was suddenly seized, on the fifth day of the disease, with a considerable oppression of respiration. He regarded this accident as a sign of inflammation of the lungs; an engorgement of the chest had taken place, he said. I proposed the application of vesicatories to the nape of the neck, or behind the ears: the patient would not consent to it, fearing an augmentation of the source of the irritation. He told me that he must be bled in the tongue, to produce a *derivation* towards the throat; accusing the bleeding in the foot, which had effected, according to him, too considerable a *revulsion*. I endeavoured in vain to persuade him of the little relation there was between the blood-vessels of the lungs and those of the tongue: I was obliged myself to open the veins of the tongue. He lost a great quantity of blood; he got weak; the chest became completely engorged; delirium came on; and the patient died on the seventh day. I made researches in modern authors about this case in vain. SYDENHAM does not specify this accident; BAGLIVI says something about it, and especially on the state of the pulse, which I found in my patient such as Baglivi describes it,—equal, soft, full, strong, and apparently good. WILLIS does not treat of angina. FERNELIUS and HOULIER only advance some generalities respecting it, as well as our later professors. BENIVENIUS, who confirmed to me on this subject what CELSUS had written, speaks favourably of scarifications: he had hit the mark, and agreeably to the spirit of the sentence of Hippocrates. My patient would not hear of it; and I may assert that his obstinacy arose from the notions he had respecting the circulation of the blood. He wished to elicit away the blood which became congested in the lungs by a *derivation* of it towards the throat: a foolish project, and dictated from ignorance of the true laws of the circulation! May I say that I have seen more than one practitioner who has talked about those laws, and who did not understand them better than my patient? They told me, coolly, that the bleeding from the arm disgorged the lungs, because the arm and the lungs received branches from the same trunk; that bleeding in the foot elicits the blood to the lungs, because the head, the lungs, and the legs, have vessels coming from the same trunk; and that, consequently, the column of blood drawn by the bleeding in the foot, falls first on the lungs, &c. What I cannot doubt is, that a transport had taken place from the throat to the lungs, in my patient, as it takes place from the interior to the outward part of the throat in some other cases,—that is, by means of the cellular tissue. It happens sometimes that, after the fall of matter on the lungs, nature regains her powers, the malady changes into peripneumony, which terminates with purulent evacuation. It is this which is indicated by the sentence of Cos. It marks out as a term, the *seventh day, in which gangrene takes place, or else suppuration is established.* It is true that Hippocrates has taken a different *term* or day, in another of his works. "*If in an angina the throat appears to be cured, and the swelling having disappeared, the disease falls on the lungs, then fever and stitch in the side appear, and the patient generally dies.* But, *if he survives* FIVE DAYS, *an abscess will be formed; at least, unless cough comes on; for then the expectoration becomes abundant, and the patient becomes convalescent.*" (De Morbis, lib. ii.) There are, then, three different cases to be distinguished in this falling of the angina. In the first, gangrene takes place on the *seventh day;* if this be passed over, suppuration is established. In the second, suppuration happens after the *fifth* day; and in the third, cough is attended with critical expectoration. This third course of the disease is undoubtedly the most favourable; it is that in which the lungs are so disposed as to reject by cough the matter of the fluxion. In the second, an abscess is formed, according to Hippocrates, on the fifth day; and in the first, gangrene is to be feared on the *seventh* day. Is there not an evident contradiction in these texts? Take care that they do not both express the same kind of disease. Sometimes the patient is suffocated in the first few days after the falling of the angina; this is when there is a gangrenous disposition, and when the lungs have already suffered considerably. Sometimes he resists *five* days, after which an abscess is formed; it is when the lungs were affected, without being in a manner impervious to the passage of the matter. Lastly, gangrene sometimes happens on the *seventh* day: this is when the peripneumony has had time to run through its stages, and to fall into gangrene, or to resist it and give rise to suppuration. Thus the different statements of Hippocrates do not contradict each other; they designate different stages of the same species of disease, which only varies in being more or less severe. It appears to me of particular importance to remark, that when the fall of the angina takes place on the lungs, there are only two distinct results,—abscess and expectoration. Now, the precursors of these two revolutions are pain and fever, which we must be careful not to attempt to remove precipitately by any violent measures. "*The pain in the side, in a patient affected with angina, which has not terminated by coction, joined with depression of strength, becomes mortal, without there being reason to doubt such a termination.*" (Coac. No. ccclxxiv.) This sentence has much analogy to the second in the book *De Arte*, 99. The angina having disappeared, and being reported cured, a collection takes place in the lungs in an insensible manner; the patient appears to become convalescent; fever is hardly sensible; a collection of matter is formed in the lungs; the strength diminishes, and the patient either falls into pulmonary consumption or dies suddenly. I have seen this accident take place in an unequivocal manner at the hospital *la Charité*, at Paris. The patient, who had an affection of the throat, and which had been treated in the ordinary way, was about to leave the hospital; he had taken leave: he had breakfasted, according to custom, on the day of departure. He died suddenly as he was going out; and the chest was found to be full of purulent matter.

I have seen many patients die after they had become apparently convalescent from sore throats, on the same day as a purgative had been taken. It once happened to one of my patients, eighteen years since. I purged an old man: I believed him to be cured of a sore throat, which had appeared to be very slight: he died during the operation of the medicine. These examples had taught me to respect the *sentence* of Hippocrates. I have learned to fear the consequences of a sort of oozing which takes place through the cellular tissue, and which gradually fills the chest, and ruins the life of the patient without our being at all aware of what is taking place. I have learned to dread the stifling, and abortion, as it were, of cynancheal inflammation, by means of bleeding and purgatives. Relapses, pulmonary consumption, or even the most unexpected occurrence of death, are sometimes the consequences of incautious treatment of this kind. I shall never believe a real angina past all danger, but when there have been unequivocal signs of what has been termed *coction.* This is worthy of the serious reflection of those who pay no attention to those sorts of critical revolutions; who are not inclined, as they say, to believe in *crises*

and *coctions.* These salutary efforts of nature sometimes happen, even contrary to the intention of those who treat the disease, or at least without their being aware of their occurrence. Nature will sometimes carry the patient safe through all the dangers excited by the most petulant mode of treatment.

"*Patients attacked with angina, and who have the throat dry and smooth, with but little spitting, are in danger.* (Coac. No. ccclxix.) *Every thing is to be feared for those patients who, being attacked with angina, do not spit concocted matter as soon as possible.* (Ib. No. ccclxxi.) *Nothing is so dangerous as the angina in which there does not appear outwardly any product of a salutary effort.*" (Ib. No. ccclxii.) Hitherto we have only considered the phenomena which succeed the fall of an angina on the lungs: these phenomena are accidents more or less to be feared. The cellular substance, embarrassed by the remains of an ill-effected coction, cannot fail running into suppuration or gangrene. The *sentences* of this article designate the revolutions necessary for the favourable termination of the angina. The applications of these sentences are evident, and the truth of them demonstrated by a multitude of observations. Every reflective physician will agree with this; and he will acknowledge that the expectoration is sometimes so abundant, that it is not possible to imagine but that it comes from the whole of the cellular tissue of the throat, the chest, and the adjacent parts, which constitute the mucous, spongy, and cellular bag, of which we have so often spoken.* It will be found, on due consideration of the subject, that all the cellular tissue of the chest, and its appurtenances, is often embued with heterogeneous matter; and the effort which all this part of the mucous system makes to evacuate it by expectoration, will be readily recognized. We may at least conclude, that those who endeavour to remove this matter by repeated bleedings and purgatives, directly oppose the natural effort of the parts, which, according to the *sentences* of Hippocrates, relieve themselves by expectoration, and should be relieved in this way; without which the consequences of the malady are much to be feared.

I have already spoken of the little resource I for a long time found in some authors who had not perceived the views of Hippocrates respecting angina; but we are now amply supplied with the desired information, by the commentator of the works of Boerhaave. Many sentences of Hippocrates are very well explained in his Commentaries. That which relates to the falling of angina is not forgotten. It is an interesting fact, that the *sentence* of Hippocrates may be considered calculated to refute the 809th proposition of Boerhaave. The following is that proposition. "We must be careful not to lose a moment in inflammatory angina: for this reason, 1°, we should have recourse to bleeding, and the bleeding should be to a considerable extent, and repeated until debility, paleness, coldness, and shrinking of the vessels, prove that the powers which remain in the patient cannot increase the swelling and the rigidity of the vessels; 2°, the bowels should be acted

---

* BICHAT's treatises on the *Membranes* and on the *Cellular System*, in his *Anatomie générale* and *descriptive*, best explain this passage.—EDIT.

on by strong purgatives, taken by the mouth and given as glysters; and these purgatives and glysters should be repeated."

In regard to this, it may be remarked, that Boerhaave does not indicate the vessels which should be opened; and the conditions which he requires in the effects of blood-letting are extremely vague: the shrinking of the vessels, is more than vague, and perhaps much to be feared. Aretæus saw many patients die under the lancet. It seems that Boerhaave inculcates the use of violent purgatives immediately after blood-letting; but is it after the first, or the repetition of that operation? He does not designate on what day of the disease this violent purgative should be again resorted to. This proposition of Boerhaave is copied from Celsus and some other authors; who, however, explain themselves with more precision and reserve. With respect to the use of violent purgatives, Hippocrates said, that "when expectoration does not appear in a favorable manner in cynanche, a great number of stools reduce the patient to a sort of paralytic state." A violent purgative will occasion the same consequences. Avicenna would not use purgatives in the commencement of angina, neither would Coelius Aurelianus. Rhases employed very gentle ones, as well as Fernelius, who did not believe them proper in purely inflammatory angina. Sydenham hesitated, as well as Sennert, when it was necessary to purge in this case. Hoffman and Zacutus Lusitanus, were decidedly in favour of gentle remedies in this malady. But, why did Boerhaave not speak of emetics in this affection in preference to purgation? since Praxagoras and Heraclitus of Tarentum, amongst the ancients; and Rivière, and many other moderns, have rescued patients from death, by making them vomit, in this disease? Ætius expressly observes, that Archigenes did not favour prompt and copious blood-letting in angina, for fear that by this means the matter might be made to fall on the lungs. Fernelius, and, before him, Trallianus, had made the same remark; this is the principal point I wish to arrive at. This reflection of Archigenes accords very well with the sentence of Hippocrates concerning the falling of the angina on the lungs. I can myself assert that I have seen the affection of the throat disappear under the use of blood-letting, and the expectoration cease; and then the lungs become oppressed, as in the physician whose case is above related.

I have witnessed equally bad effects from the use of violent purgatives. "I have often seen," says Van Swieten, "that on the disappearance of the pain of the angina, the lungs became embarrassed, sometimes with pain in the side: many have died, and but very few have recovered, amongst those whom I have treated; although I very promptly employed the most efficacious remedies." If these remedies were, as there is reason to believe, those indicated by Boerhaave, does not the acknowledgment of Van Swieten render the propriety of those remedies very liable to doubt? What worse would have happened from proceeding a little less violently? An emetic given at a proper time might have removed the obstacles to the natural course of the disease, and have favoured its *maturation*. It is a fact of which I believe every French physician would be able to produce proofs. Every one should be content to say what he has observed. I remember that, in my younger days, my father (ANTOINE DE BORDEU) many times restored hope and calmness to whole villages, where epidemic sore throats had been making cruel ravages. An emetic was one of his principal resources. The following is a short account of what I have myself witnessed. 1. In 1744 and 1745, in *le Béarn*, my country, sore throat was very prevalent, and great numbers died from it. I preserved many of those I treated, even when they appeared to have arrived at the last extremity, by the use of emetics. 2. In 1745 and 1746, at Montpellier, an epidemic sore throat, in which I saw emetics boldly exhibited to all ages and both sexes, and in the most inflammatory anginas. In 1747 and 1749, at Paris, and at the Royal Infirmary at Versailles, where it was treated as at Montpellier, only at Paris more dependance was placed on bleeding than at Montpellier. 4. Similar cases at Paris, especially in 1758, 1759, and 1762; during the epidemics called by some persons the *drollette*, and afterwards the *petite poste*. I then witnessed an affection of the throat, but slight in the first instance, go on constantly increasing to the fourth day, and then terminate in death, after seven bleedings; a critical evacuation by the nose and ears, after two bleedings and three purgatives had been used without advantage; but a fourth purgative produced convulsions and death. In a convent where I was called, with some other physicians who consented to use emetics, the beneficial efficacy of them was decisively evident. If we may not be permitted to abandon (in sore throat, as well as in many other diseases), three-fourths of the task to nature, it appears to me that there would be less inconvenience arise from persisting in the use of emetics, than from bleedings and purgatives, especially strong purgatives: for I am well assured that many drinks and potions commonly used and regarded as purgatives, are merely sorts of *indifferent* diluents, not producing any important effects in many cases. It must not be dissimulated, that the purgative prescribed for angina by Hippocrates, Galen, and Trallianus, was of a violent kind; it was *elaterium:* but it is known that this drug acts as a powerful emetic, and it could not fail to excite vomiting in angina, on giving it to the extent of four, six, and even twelve, grains, which were the ordinary doses, accorcing to Dioscorides. If, then, Boerhaave and Van Swieten rely on the authority of Hippocrates, in giving strong purgatives, because he employed *elaterium*, we can also look to the same authority for the use of emetics.*

---

* The above observations rest on some arguments used by Bordeu to show that purgatives favour the infiltration of the matter formed about the throat, through the cellular tissue, into the lungs: whilst emetics tend to produce the contrary effect. He brings forward a multitude of pathological observations analogous to those related at the commencement of the above extract, tending to show that this mode of transmission of fluids from one part of the body to the other, and the dependance of this transmission on vital efforts, are facts which have been too much neglected, from our disposition to attribute every thing of the kind to circulation in vessels. But here we terminate our series of extracts from the works of this great physician.—EDITOR.

